# Organoid and Enteroid Modeling of *Salmonella* Infection

**DOI:** 10.3389/fcimb.2018.00102

**Published:** 2018-04-04

**Authors:** Yuebang Yin, Daoguo Zhou

**Affiliations:** ^1^Department of Gastroenterology and Hepatology, Erasmus MC-University Medical Center, Rotterdam, Netherlands; ^2^Key Laboratory of Molecular Microbiology and Technology, Ministry of Education, TEDA Institute of Biological Sciences and Biotechnology, Nankai University, Tianjin, China; ^3^Department of Biological Sciences, Purdue University, West Lafayette, IN, United States

**Keywords:** *Salmonella*, infection models, enteroids, intestine, organoids

## Abstract

*Salmonella* are Gram-negative rod-shaped facultative anaerobic bacteria that are comprised of over 2,000 serovars. They cause gastroenteritis (salmonellosis) with headache, abdominal pain and diarrhea clinical symptoms. Salmonellosis brings a heavy burden for the public health in both developing and developed countries. Antibiotics are usually effective in treating the infected patients with severe gastroenteritis, although antibiotic resistance is on the rise. Understanding the molecular mechanisms of *Salmonella* infection is vital to combat the disease. *In vitro* immortalized 2-D cell lines, *ex vivo* tissues/organs and several animal models have been successfully utilized to study *Salmonella* infections. Although these infection models have contributed to uncovering the molecular virulence mechanisms, some intrinsic shortcomings have limited their wider applications. Notably, cell lines only contain a single cell type, which cannot reproduce some of the hallmarks of natural infections. While *ex vivo* tissues/organs alleviate some of these concerns, they are more difficult to maintain, in particular for long term experiments. In addition, non-human animal models are known to reflect only part of the human disease process. Enteroids and induced intestinal organoids are emerging as effective infection models due to their closeness in mimicking the infected tissues/organs. Induced intestinal organoids are derived from iPSCs and contain mesenchymal cells whereas enteroids are derive from intestinal stem cells and are comprised of epithelial cells only. Both enteroids and induced intestinal organoids mimic the villus and crypt domains comparable to the architectures of the *in vivo* intestine. We review here that enteroids and induced intestinal organoids are emerging as desired infection models to study bacterial-host interactions of *Salmonella*.

## General introduction of *Salmonella*

*Salmonella* are Gram-negative rod-shaped and facultative anaerobes belong to the family of *Enterobacteriaceae* (Coburn et al., 2007). According to the recent classification by the International Code of Nomenclature of Bacteria, the genus *Salmonella* are classified into two distinct species including *Salmonella enterica* and *Salmonella bongori* based on their 16S rRNA sequence relatedness (Popoff et al., 2003). *Salmonella bongori* (V) is treated as a separate species due to its unique clinical features (Fierer and Guiney, 2001). *Salmonella enterica* is further classified into six subspecies: *enterica* (I), *salamae* (II), *arizonae* (IIIa), *diarizonae* (IIIb), *houtenae* (IV), and *indica* (VI), mainly based on their genomic sequence and biochemical properties (Fierer and Guiney, 2001). Diverse biochemical properties of the flagellar, carbohydrate and lipopolysaccharide (LPS) of *S. enterica* divided them further into over 2,000 serovars (Eng et al., 2015). Over 50% of these serovars belong to *S. enterica* subspecies enterica which are responsible for most infections in human (Itri et al., 2017). *Salmonella* are also classified based on their somatic (O), capsular (K), and flagellar (H) antigenic determinants (Brenner et al., 2000). The commonly used *Salmonella* classification in clinical laboratories is based on simple agglutination reactions to antibodies or antisera specific to the somatic O antigens containing six serogroups designated A, B, C1, C2, D, and E (Eng et al., 2015).

*Salmonella* infections remain a big burden for the public health worldwide (Eng et al., 2015). There are three main types of Salmonellosis: (1) localized intestinal infection (gastroenteritis), (2) systemic infection of otherwise healthy hosts (typhoid), and (3) systemic infection of immune-compromised hosts (Griffin and McSorley, 2011; Hurley et al., 2014). *Salmonella* strains that cause these infections are separated into typhoid *Salmonella* and non-typhoid *Salmonella* (NTS) based on the clinical patterns in human salmonellosis (Crump et al., 2015; Eng et al., 2015). Typhoid *Salmonella* infection seems to be more severe, in which patients show prodromal symptoms such as headache, abdominal pain and diarrhea (or constipation), followed by the onset of fever, which might sustain an incubation period of 1 week or more (Eng et al., 2015). Typhoid *Salmonella* strains are normally restricted to humans causing typhoid fever (also called enteric fever), while NTS strains have a broader host-range and represent zoonotic features (Gordon, 2011). The typhoid *Salmonella* infections occur mostly in developing countries including many regions of the African and Asian continent. The illness causes 93.8 million foodborne cases and 155,000 deaths annually (Eng et al., 2015). In comparison, gastroenteritis is caused mainly by *S. enterica* Serovar Typhimurium (*Salmonella* Typhimurium) and Serovar Enteritidis, which are common even in developed countries (Eng et al., 2015). The clinical signs of gastroenteritis can be diarrhea, cramping, and most patients usually recover within 4–7 days without treatment (Griffin and McSorley, 2011). Salmonellosis is transmitted mainly via food or water contaminated with human or animal feces (Crump et al., 2015). NTS have been reported to transmit via contaminated animals and animal products (Crump et al., 2015). The mortality caused by typhoid *Salmonella* strains can be up to 7% even when antibiotics are used (Eng et al., 2015). The incidence of enteric fever in the USA and European countries is normally low, representing less than 10 per 100,000 each year (Eng et al., 2015). In contrast, the invasive infections caused by NTS are estimated to be 3.4 million with 681,000 deaths worldwide in 2010 (Crump et al., 2015). Up to 57% of these illnesses and deaths are in Africa (Crump et al., 2015). Most NTS infections occur in animals, while it can also happen in infants, young children, elderly people and immunocompromised patients (Eng et al., 2015). The actual cases of *Salmonella* infections are estimated to be much higher than the reported numbers because milder cases are likely not diagnosed or reported, especially in some developing countries (Hurley et al., 2014).

Many assays have been developed to diagnose *Salmonella* infections. Widal test is the most commonly used diagnostic assay based on agglutinating antibodies against *Salmonella* LPS (O) and flagella (H). The enzyme-linked immunosorbent assays (ELISAs) and sodium dodecyl sulfate-polyacrylamide gel electrophoresis (SDS-PAGE) immunoblotting assays are useful in measuring antibodies in patients' sera. Recently, PCR, real-time PCR, and proteomic approaches have been used to analyze bacterial genes and proteins (Kumar et al., 2012; Nigro et al., 2016). Once the infection is diagnosed, antibiotics might be used to treat infected patients with severe gastroenteritis, while most milder infections do not require antibiotics treatment (Boyle et al., 2007; Hurley et al., 2014). Two typhoid vaccines have been licensed against enteric fever, both are only suitable for endemic situation (Griffin and McSorley, 2011; Crump et al., 2015; Eng et al., 2015). Vaccines against NTS infections have been developed recently (Griffin and McSorley, 2011; Eng et al., 2015). Currently, food safety from farm to fork and treatment of municipal water remain the most effective measures to control the transmission of *Salmonella* (Crump et al., 2015).

The virulence determinants needed for *S*. Typhimurium are similar to those of many other intestinal pathogens: First, it needs to successfully survive the hostile acidic environment in the stomach before making its way to colonize the small intestine. In the intestine, the bacteria must breach the barrier of intestinal epithelial cells and it has to survive inside the host cells. Pathogenic *Salmonella* spp. evolve complex systems, which enable the organisms to respond and survive in the stomach with low-pH (Foster, 1995), and to reach M cells and enterocytes in the small intestine (Takeuchi, 1967; Moulder, 1985; Lindquist et al., 1987; Nietfeld et al., 1992; Clark et al., 1994, 1996; Jones et al., 1994; Sansonetti and Phalipon, 1999). *Salmonella* Typhimurium has the ability to enter non-phagocytic eukaryotic cells and to exist as intracellular parasites inside enclosed vacuoles (Takeuchi, 1967; Moulder, 1985). The intracellular environment provides a unique niche for the bacteria to multiply and evade host immune responses. In addition, *S*. Typhimurium is capable of surviving and replicating within macrophages (Buchmeier and Heffron, 1989).

Several studies have led to the identification of genes that are required for *Salmonella* pathogenesis, in particular for *Salmonella* invasion into non-phagocytic cells (Galán and Curtiss, 1989; Ochman et al., 1996; Shea et al., 1996; Blanc-Potard and Groisman, 1997; Wong et al., 1998; Wood et al., 1998). Many of these virulence genes and operons are located in large genetic elements of the *Salmonella* chromosome. Since these large elements are absent from the chromosome of closely related *Escherichia coli*, they are termed pathogenicity islands. Virulence plasmids also contribute to *Salmonella* survival in macrophages and virulence (Gulig, 1990; Guiney et al., 1994; Wallis et al., 1995). At least five pathogenicity islands have been identified in *Salmonella* (Galán and Curtiss, 1989; Ochman et al., 1996; Shea et al., 1996; Blanc-Potard and Groisman, 1997; Wong et al., 1998; Wood et al., 1998) that contribute to virulence at defined stages of the infection process. *Salmonella* Pathogenicity Island I (SPI1) is the best studied one. It is located at centisome 63 on the *Salmonella* chromosome and is 43-kb in length. SPI1 is required for *Salmonella* entry into M cells (Clark et al., 1996) and epithelial cells (Galán and Curtiss, 1989) of the intestine. This is consistent with the fact that SPI1 mutants are defective in virulence when administered orally, but not if given systematically (Galán and Curtiss, 1989). Mutants that are defective in entry into epithelial cells were found to be avirulent in studies using the mouse-typhoid model (Galán and Curtiss, 1989) and in calves (Watson et al., 1998; Tsolis et al., 1999, 2000). SPI2, SPI3, and SPI4 are situated at centisome 31, 82, and 92 of the *Salmonella* chromosome, respectively. Genes in these three islands are essential for *Salmonella* survival and growth in the host (Hensel et al., 1997, 1998; Shea et al., 1999; Vazquez-Torres et al., 2000). SPI5 was originally found to be involved in inflammation and fluid secretion in the intestine (Norris et al., 1998; Wood et al., 1998). We have shown that at least one gene in this island (*sopB*) is also involved in the *Salmonella* invasion process (Galan and Zhou, 2000).

SPI1 and SPI2 encode specialized protein secretion and translocation systems termed type III secretion system. Genes in SPI1 can be divided into three groups: (1) one includes genes that encode the actual secretion/translocation apparatus; (2) a second group encodes proteins that are secreted and/or translocated into host cells; (3) a third group involves in gene regulation. The SPI1 secretion apparatus was shown by electron microscopy (Kubori et al., 1998) that appears to constitute a “needle complex” that is similar to the bacterial flagella system both biochemically and structurally. Purified needle complexes consist of at least three proteins encoded in SPI1 (PrgK, PrgH, and InvG). Mutations in *prgK, prgH*, or *invG* have been shown to abolish the secretion of a panel of *S*. Typhimurium proteins (SipA, SipB, SipC etc.). The translocation of these bacterial proteins into eukaryotic host cells is required for *Salmonella* invasion into non-phagocytic epithelial cells. Secretion has been reported to require host-cell contact (Zierler and Galán, 1995). However, these proteins are secreted under certain laboratory conditions in sufficient amounts to facilitate their studies in the absence of host cells. These secreted proteins can be visualized by SDS-PAGE from supernatants of *S*. Typhimurium cultures under such inducing conditions. At least nine secreted proteins have been identified using this approach, including: AvrA, SipA, SipB, SipC, SipD, SopE, SopE2, SopB, and SptP. During the infection process, these proteins are thought to be translocated inside the host cell, where they engage host cell components to promote bacterial uptake (Galán, 1998, 1999).

Successful *Salmonella* infection requires the bacteria to gain a growth advantage over the intestinal microflora while inducing intestinal inflammation. Although it remains a challenge to understand how *Salmonella* achieve this in the gut, several recent studies have shed light on the molecular mechanisms that *Salmonella* use. It has been reported that the long O-antigen chain in *Salmonella* conferred a growth advantage in the mouse colitis model (Crawford et al., 2012). It was proposed that *Salmonella*-induced colitis increased the luminal concentrations of total bile acids and *fepE*-mediated (O-antigen assembly) bile acid resistance is responsible for conferring a fitness advantage during luminal growth in the inflamed intestine (Crawford et al., 2012). Furthermore, in search for additional signals generated during *Salmonella*-induced inflammation, the methyl-accepting chemotaxis proteins (MCPs) including Trg, Tsr, and Aer, were identified to enhance the fitness of *Salmonella* in a mouse colitis model (Rivera-Chávez et al., 2013). Thus, it is becoming apparent that *Salmonella* utilize their virulence factors to induce inflammation and to generate inflammation-derived nutrients to edge out competing microbes in the inflamed intestine (Rivera-Chávez and Báumler, 2015). Furthermore, *Salmonella* are capable of using inflammation-derived nitrate to respire anaerobically and to compete with the commensal microbes in the gut using three nitrate reductases, encoded by the *narGHI, narZYV*, and *napABC* genes (Lopez et al., 2015). A recent study also demonstrated that the disturbance of the commensal *Clostridia* increased the susceptibility to *Salmonella* infection in a mouse model (Rivera-Chávez et al., 2016). This is largely due to the decreased butyrate levels, produced from the butyrate-producing Clostridia, led to increased oxygenation in the gut, promoting the aerobic expansion of *Salmonella* (Rivera-Chávez et al., 2016).

## Current models for studying *Salmonella*

Many experimental models have been developed to study *Salmonella* infections including various *in vitro, ex vivo*, and *in vivo* models (Table 1). The most commonly used ones are the two-dimensional (2-D) immortalized cell line models including the Caucasian colon adenocarcinoma (Caco2) cells (Martinez-Argudo and Jepson, 2008), the immature human normal fetal intestinal epithelial cells (H4) (Newburg et al., 2016), the mature human metastatic colonic epithelial cells (T84) (Newburg et al., 2016), the human normal colon mucosal epithelial cells (NCM-460) (Newburg et al., 2016), the Microfold cells (or M cells) (Martinez-Argudo and Jepson, 2008), the RAW 264.7 murine macrophage cells (Tang et al., 2012), the cervical cancer (HeLa) cells (Fang et al., 2017), and the gut fermentation models (Le Blay et al., 2009). In addition, three-dimensional (3-D) organotypic models derived from the Int-407 cell line (later found to be a HeLa derivative) and the human colorectal adenocarcinoma cells (HT-29) (Nickerson et al., 2001; Höner Zu Bentrup et al., 2006). Salmonellosis in humans, monkeys, and calves are known to affect primarily the distal ileum and the proximal colon (Kinsey et al., 1976; Giannella et al., 1977; Wray and Sojka, 1978; McGovern and Slavutin, 1979; Samuel et al., 1980). These models have greatly aided the genetic, cell biological and biochemical analysis of the infection process.

**Table 1:** Salmonella infection models.

	**Year**	**Author**	***Salmonella* type**	**Model**
*In vitro*	2001	Nickerson et al.	*Salmonella* Typhimurium	3D organotypic model based on the human embryonic intestinal epithelial cells (Int-407) (Barrila et al., 2010)
	2006	Zu Bentrup et al.	*Salmonella* Typhimurium	3D organotypic model based on the human colon adenocarcinoma cell line (HT-29 cell line) (Höner Zu Bentrup et al., 2006)
	2008	Isabel Martinez-Argudo and Mark A. Jepson	*Salmonella enterica*	M cell model (Martinez-Argudo and Jepson, 2008)
	2009	Le Blay et al.	*Salmonella* Typhimurium	Colonic fermentation model (Le Blay et al., 2009)
	2012	Tang et al.	Clinical non-typhoid *Salmonella* (NTS) isolates	RAW 264.7 murine macrophage cell line (Tang et al., 2012)
	2014	Dostal et al.	*Salmonella* Typhimurium	Gut fermentation-cell model (Dostal et al., 2014)
	2014	Zhang et al.	*Salmonella* Typhimurium	Crypt-derived mouse intestinal organoids (Zhang K. et al., 2014)
	2015	Forbester et al.	*Salmonella* Typhimurium	Intestinal organoids derived from human induced pluripotent stem cells (hIPSCs) (Forbester et al., 2015)
	2016	Newburg et al.	*Salmonella* Typhimurium	Immature human normal fetal intestinal epithelial cell (H4), mature human metastatic colonic epithelial cell (T84) and human normal colon mucosal epithelial cell (NCM-460) (Newburg et al., 2016)
	2017	Fang et al.	*Salmonella* Typhimurium	HeLa cells, Caco-2 cells, THP-1 cells and LS174T cells (Fang et al., 2017)
*ex vivo*	1997	Frost et al.	*Salmonella* Typhimurium	Calf ileal epithelium (Frost et al., 1997)
	2004	Haque et al.	*Salmonella* Typhimurium TML	Human intestinal *in vitro* organ culture (IVOC) (Haque et al., 2004)
	2012	Tsilingiri et al.	*Salmonella* Typhimurium	Organ culture model (intestinal mucosa) (Tsilingiri et al., 2012)
	2015	Boyle et al.	*Salmonella* Typhimurium	Perfusion of the isolated rat small intestine (Boyle et al., 2015)
	2016	Newburg et al.	*Salmonella* Typhimurium	Immature human intestinal tissue (Newburg et al., 2016)
*In vivo*	1973	Giannella et al.	*Salmonela* Typhimurium	The ligated rabbit ileal loop model (Giannella et al., 1973)
	2003	Barthel et al.	*Salmonella* Typhimurium	C57BL/6 mice (Barthel et al., 2003)
	2007	Woo et al.	*Salmonela* Typhimurium	SLC11A1 wild type mice (Woo and Berk, 2007)
	2009	Ren et al.	*Salmonella* Typhimurium	C57BL/6 mice (Ren et al., 2009)
	2011	Mian et al.	*Salmonella* Typhi	Humanized mice (alymphoid RAG-2-/-γc-/- mice engrafted with human leukocytes) (Firoz Mian et al., 2011)
	2012	Özkaya et al.	*Salmonella* Typhimurium	BALB/c mice (Özkaya et al., 2012)
	2012	Mathur et al.	*Salmonella* Typhi	A mouse model (tlr11-/+ mice) (Mathur et al., 2012)
	2014	Zhang et al.	*Salmonella* Typhimurium	Neonate mice (Zhang Y. G. et al., 2014)

*In vitro* cell culture lines are relatively easy to maintain and provide a more consistent environmental niche for evaluating bacterial survival and replication than most animal hosts (Finlay and Brumell, 2000). Genetic manipulations in these cell lines greatly aided the investigation of how *Salmonella* interact with host epithelial and macrophage cells (Finlay and Brumell, 2000; Zhou, 2001, 2006; Zhou and Galán, 2001). However, immortalized cell lines lack the complexity of cell types and the robust immune components, thus cannot closely mimic the natural infection process (Finlay and Brumell, 2000). For instance, the apoptosis process is regulated differently in immortalized cells comparing to that of healthy tissues (Finlay and Brumell, 2000). In addition, many mammalian cells cannot sustainably maintain their original characteristics during the long culturing process (Finlay and Brumell, 2000). For example, derivative cells may arise (Foulke-Abel et al., 2014). The 2-D cultures of immortalized cells only have one cell type, making it difficult to mimic complex architecture in the mucosa *in vivo* (Yin et al., 2015).

The lack of suitable models for testing effects of antimicrobials on enteropathogens hampers the development of novel antimicrobials combating *Salmonella* infections. Commonly used animal models cannot reproduce microbiota residing in the human intestine, and most continuous models for human intestinal microbiota have limitations on the microbial diversity, stability, cell density, and lack the ability to support long-term studies. An *in vitro* continuous colonic fermentation model has been developed to allow the bacteria to grow in biofilm structures, facilitating tests of new antimicrobials against *Salmonella* infections (Le Blay et al., 2009). This model uses immobilized child fecal microbiota and the introduction of *Salmonella* for the proximal colon to produce high bacterial density in gel beads and in reactor effluents. The growth conditions allow the protection of sensitive bacteria from shear, oxygen stress, and limit the washout and loss of less competitive bacteria. Le Blay et al. successfully used this model to examine the effects of two antibiotics on *Salmonella* and on the dynamic change of microbiota. Their result is consistent with *in vivo* data validating the fermentation model as a promising model platform for development of new antimicrobials against *Salmonella*.

Fully differentiated, functional intestinal epithelia *in vivo* possess unique organization of junctional, extracellular matrix, and brush border proteins, as well as highly localized mucin production (Höner Zu Bentrup et al., 2006). To mimic the 3-D architectural organization of the intestinal epithelia, a 3-D organotypic model has been developed to better recapitulate the characteristics associated with intestinal epithelia *in vivo* (Nickerson et al., 2001; Höner Zu Bentrup et al., 2006). This organotypic model uses RWV bioreactor based monolayer cultures of Int-407 or HT-29 cells. In contrast to the 2-D cells, the 3-D organotypic model has better organization of junctional, extracellular matrix, brush-border proteins, and highly localized mucin production (Höner Zu Bentrup et al., 2006). However, the 3-D organotypic model do not contain niches of the normal stem cells, which are responsible for renewing the intestinal tissues. To circumvent these shortcomings, *ex vivo* tissue culture models including the calf ileal epithelium model (Frost et al., 1997), the human intestinal *in vitro* organ culture (IVOC) model (Haque et al., 2004), the *ex vivo* intestinal mucosa model (Tsilingiri et al., 2012), and the *ex vivo* immature human intestinal tissue model (Newburg et al., 2016) have been developed to more closely mimic the surroundings of the organ during infection (Table 1). Despite the close resemblance of these *ex vivo* models to the clinical situation in the gut, their short lifetimes, laborious setups, wide experimental variabilities, limited availability of cells, and limited numbers of cells have hampered their potential use in the study of *Salmonella* infections (Höner Zu Bentrup et al., 2006).

Animal models are often used to explore the virulence mechanisms of *Salmonella* infections (Santos et al., 2001). Animals possess the complex cell types, architectural organizations, and specialized organ structures. More importantly, the intact immune systems of the animals have obvious advantages over all other models and therefore are considered the closest to clinical settings over *in vitro* cell or *ex vivo* organ and tissue models. C57BL/6 (Barthel et al., 2003; Ren et al., 2009) and BALB/c mice (Özkaya et al., 2012) are most commonly used for *Salmonella* infections. The rabbit ligated ileal loop model is used as an *in vivo* model for studying *Salmonella* infections (Giannella et al., 1973). These non-human animal models, including primates, only partially mirror the human disease process due to their inherent differences from humans (Hurley and McCormick, 2003; Firoz Mian et al., 2011). To circumvent this limitation, “humanized” mice have been developed as alternative platforms to study human infectious diseases (Legrand et al., 2009). These mice are transplanted with human cells or tissues representing confined human environments suitable for infectious agents. For example, humanized mice were generated by engrafting human hematopoietic stem cells into immunocompromised mice. These mice were used to study *S. enterica* Serovar Typhi which is usually restricted to infect only humans (Firoz Mian et al., 2011). However, the high cost of humanized mice hampers its wide application.

## Primary enteroids and intestinal organoids

**Figure 1:**
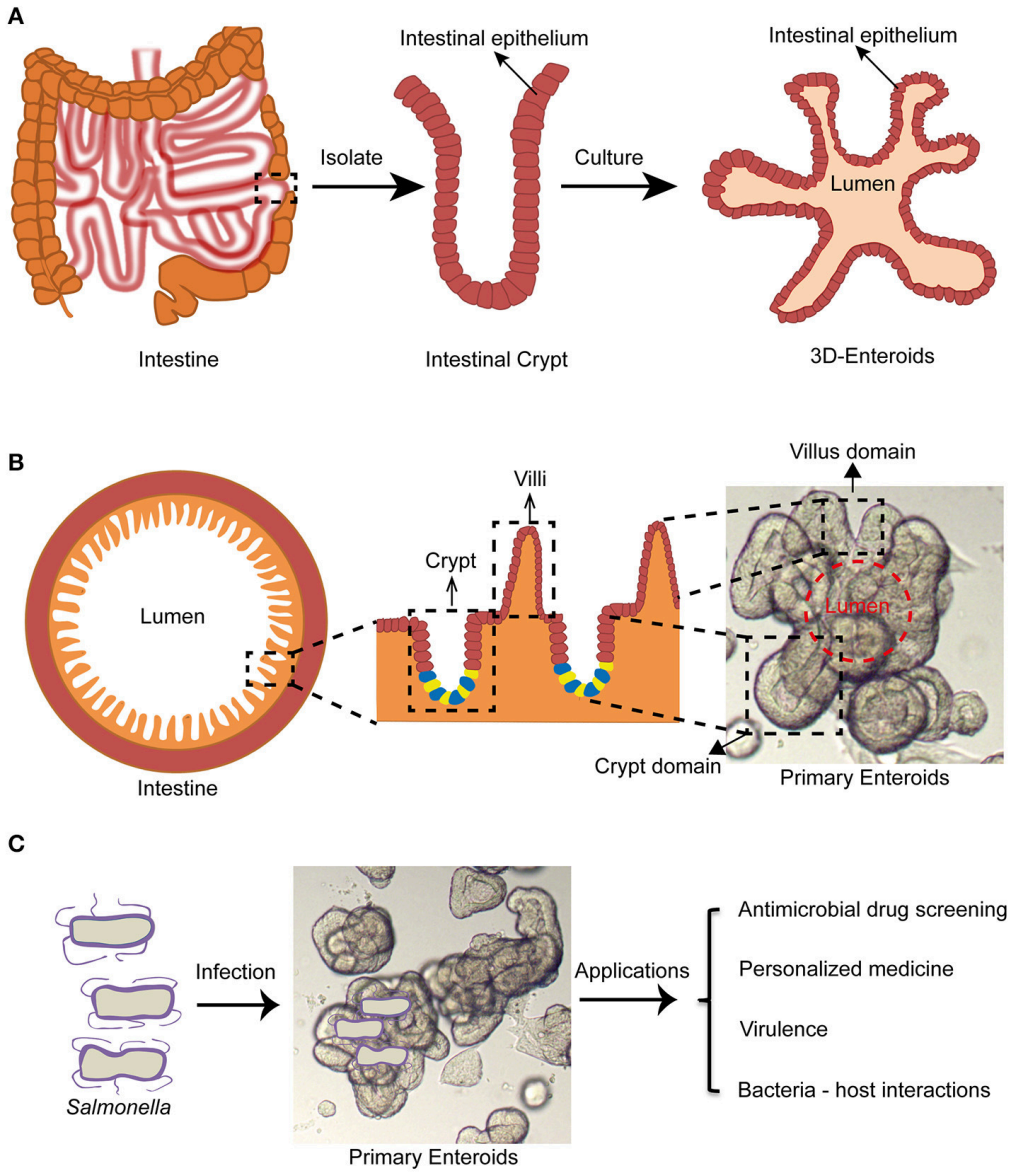
Enteroid model and its potential application in studying *Salmonella* infections. **(A)** Intestinal crypts can be isolated from surgery sections or biopsies, followed by culturing into 3-D enteroids. **(B)** Enteroids recapitulate architectures of healthy intestine containing villus and crypt domains. **(C)** Enteroids may be a promising experimental model for studying *Salmonella* infections for antimicrobial drug screening, personalized medicine, virulence mechanisms and bacterial-host interactions.

The term “enteroids” refer to multilobulated structures with a lumen that develops from intestinal stem cells (cycling crypt base columnar cells and quiescent stem cells) near the bottom of the intestinal crypts (also termed the intestinal stem cell niche), or single intestinal stem cells by formation of budding crypts (Stelzner et al., 2012). They generate the *in vivo* architecture and multi-lineage differentiation of the original intestinal epithelium in mammals (Dutta et al., 2017). They were first generated from mouse intestinal stem cells by Drs. Clevers and Sato at the Hubrecht institute (Utrecht, Netherlands) (Sato et al., 2009). Subsequently, human enteroids were successfully cultured by the same group (Sato et al., 2011). The growth of these intestinal stem cells is regulated mainly by Wnt, Notch, epidermal growth factors (EGFs), and the bone morphogenetic proteins (BMPs) signaling pathways *in vivo* (Sato and Clevers, 2013). Wnt and Notch signaling pathways play major roles in the proliferation of stem cells. EGF signals exert the robust mitogenic effects on stem cells via their corresponding receptors (EGFRs). BMP has an inhibitory effect on the stemness. Noggin promotes crypt like structures to form along the flanks of the villi (Sato and Clevers, 2013). To generate enteroids, the intestinal crypts containing the intestinal stem cell niches are separated from intestinal tissues by EDTA treatment. These crypts are then embedded in Matrigel, followed by supplementing with stemness supporting factor cocktails such as EGF, R-spondin-1, Noggin, and Wnt3a. The crypts will gradually develop into 3-D enteroids displaying many important organizations of the normal intestinal epithelium (Figures 1A,B).

Enteroids have many advantages over traditional cell culture models. For example, they can be ever-expanding, and retain their original organ identity (Sato and Clevers, 2013). Karyotypings are usually done for long term cultures and demonstrated genetic stability of the enteroids after more than 15 generations (Yin et al., 2014). Enteroids also contain luminal layers with crypt and villus domains similar to the real intestine (Figure 1B). They contain almost all intestinal epithelial cell types including the intestinal stem cells, Paneth cells, Goblet cells, enteroendocrine cells, and enterocytes (Sato and Clevers, 2013). It was reported that certain specific intestinal cell types such as tuft cells or Peyer's patch M cells can be differentiated from intestinal stem cells in the enteroids by supplementing the media with rIL-4/rIL-13 or Tnfsf11 (RankL), respectively (de Lau et al., 2012; Gerbe et al., 2016).

Induced intestinal organoids with a lumen resembling the intestine could also be generated from pluripotent stem cells (PSCs) under specific culture conditions (Stelzner et al., 2012; Dutta et al., 2017). Induced intestinal organoids from PSCs have intestinal epithelium and mesenchyme (**Figure 3**). Meanwhile, induced intestinal organoids contain most cell types that are present in the human intestine (i.e., the polarized monolayer of epithelial cells with clear apical and basal sides, microvilli, Paneth, goblet, and enteroendocrine cells, etc.) with villi domain and crypt domain (Forbester et al., 2015). It takes a few weeks for induced intestinal organoids to “mature” (Yin et al., 2015). In contrast, enteroids derived from adult stem cells (ASCs) need only several days to grow into a 3-D structure with remarkable villi domains and crypt domains resembling the *in vivo* intestinal tissue (Yin et al., 2015). In addition, primary enteroids are often taken from individuals with underlying pathological conditions. Usually, the “healthy” tissues adjacent to the diseased tissue may carry pathological changes and influence the outcome of infections.

**Figure 2:**
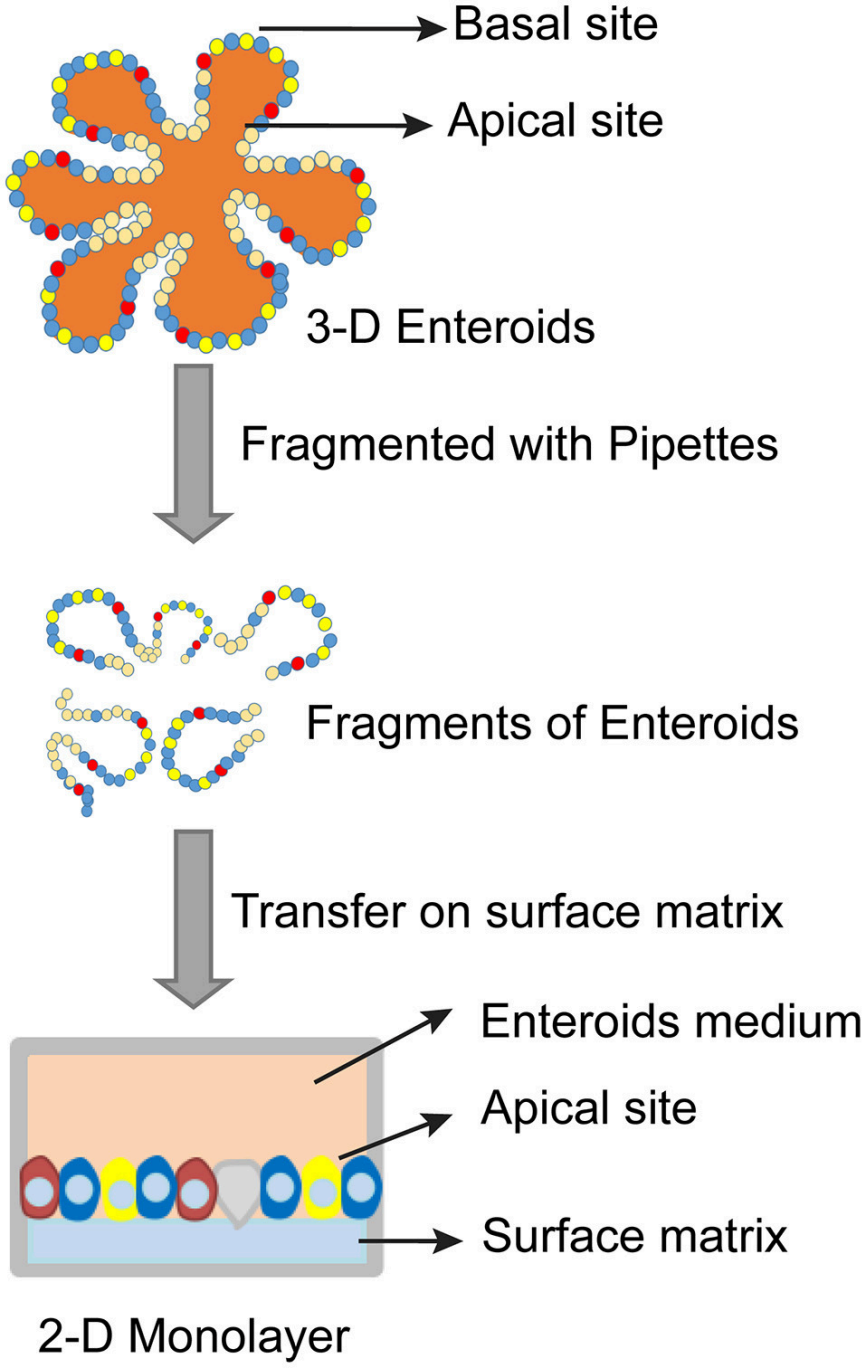
Schematic diagram of establishing 2-D monolayer enteroids.

The 3-D architecture of enteroids is believed to mimic that of an intact tissue *in vivo*. Typical 3-D enteroids develop both the apical side and basolateral side situated properly toward the lumen and the outside of enteroids (Figure 2). This organization poses a challenge to deliver the infecting bacteria to the lumen from outside of enteroids (In et al., 2016). In most cases the main site of infection is the polarized epithelium lining the intestinal lumen (Martinez-Argudo and Jepson, 2008). However, the 3-D architecture of the enteroids limited access of pathogens to the luminal epithelial surface. To overcome this, microinjection has been used to deliver enteric pathogen (e.g., mouse adenovirus 2, MAdV-2) to the lumen of enteroids (Wilson et al., 2017). Despite its remarkable resemblance to tissues/organs, enteroids can be experimentally manipulated similar to classical cell lines. These include PCR, qPCR, Western blot, immunohistochemistry (IHC), lentivirus transduction, and CRISPR/Cas9 gene editing (Miyoshi and Stappenbeck, 2013; Van Lidth de Jeude et al., 2015; Yin et al., 2015; Driehuis and Clevers, 2017). This advantage has facilitated the rapid application of enteroids.

3-D enteroids can be substituted with 2-D monolayers on top of membranes inside transwells due to the obvious time and cost considerations. In this setup, transwells provide access to the apical surfaces of 2-D monolayers (Figure 2) (Wang et al., 2017). The 2-D monolayers have been widely used to study host-pathogen interactions in a more controllable and reproducible manner comparing to that of 3-D enteroids (Wang et al., 2017). However, 2-D cultures can be maintained for only a short period of time and are suitable for studying initial pathogen/host interactions that last a few hours. In contrast, 3D cultures are better suited for examining long-term host-pathogen interactions. In addition, 3D cultures are uniquely suited for modeling the contribution of the lumen (e.g., anti-microbial response, reactive oxygen species, etc.), since they have been shown to tolerate bacteria for days without obvious tissue damage (Wilson et al., 2017).

## Enteroids and intestinal organoids for studying *Salmonella* infection

*Salmonella* infection is a dynamic process in which the bacteria encounter many cell types and organs. Proper *in vitro* models are essential for unraveling the pathogenic determinants functioning at various stages of the infection, and for developing new antimicrobials against *Salmonella* infections (Le Blay et al., 2009). Certain *Salmonella* serovars are restricted to human hosts or cause different diseases depending on whether infecting animals or humans. One such example is *Salmonella* Typhimurium (Forbester et al., 2015), which causes gastroenteritis in humans, but typhoid-like disease in mice, making it complicated to interpret data obtained from mouse experiments. Although many cell lines have been established to study *Salmonella*, the cancer-derived models cannot recapitulate the complex architecture of the intestine and might have different physiological characteristics compared to normal tissues.

**Figure 3:**
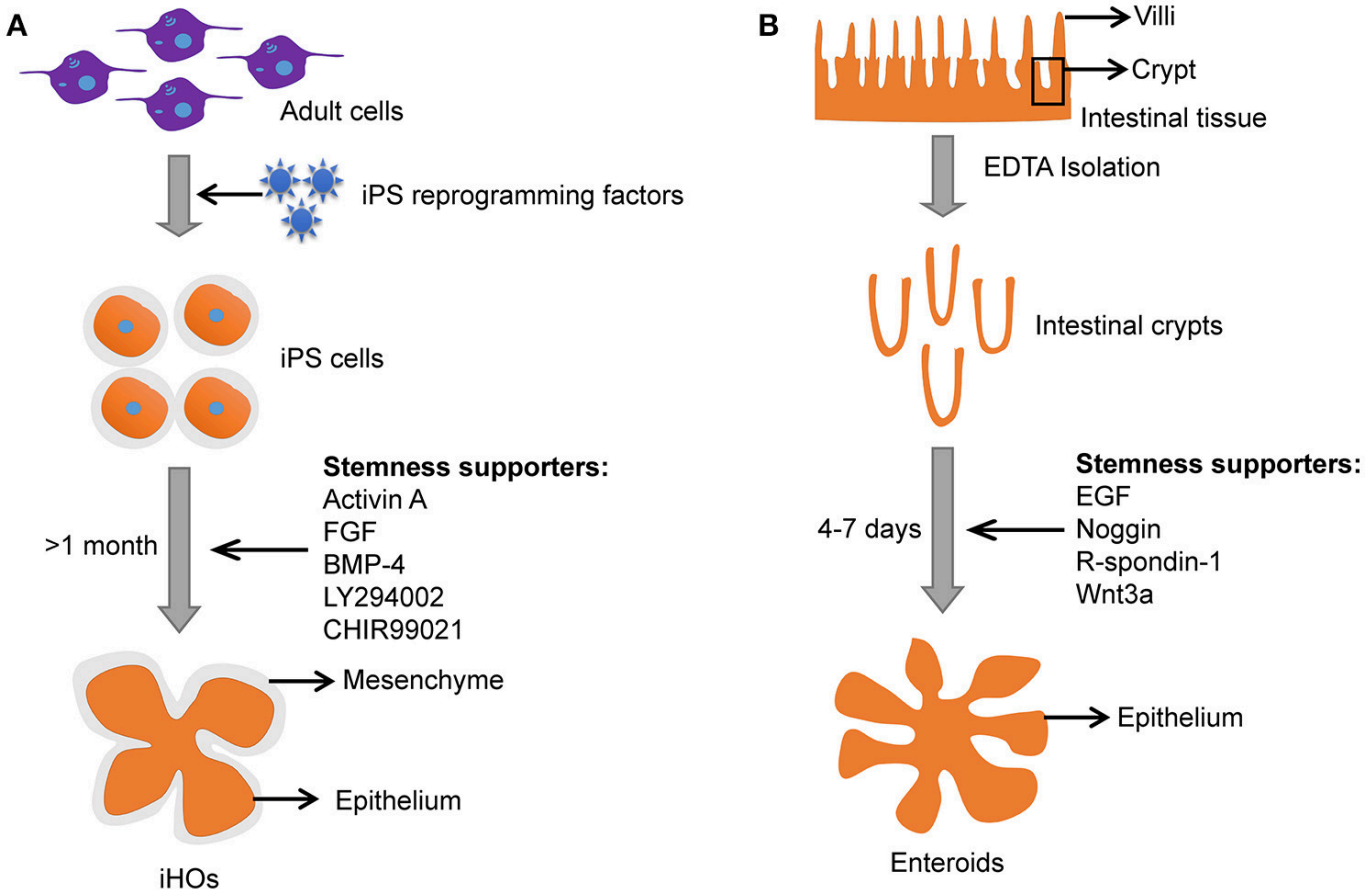
Comparison between iHOs and primary enteroids. **(A)** Culture process and features of iHOs. **(B)** Culture process and features of primary enteroids.

The iHOs have been demonstrated to be a promising infection model for *Salmonella*, and microinjection is usually used to inoculate *Salmonella* into the lumen of iHOs (Forbester et al., 2015). The iHOs need 1–2 months to “mature” before it is suitable for experimentation and the 3-D epithelial structure is surrounded by mesenchyme (Figure 3; Yin et al., 2015). In contrast, enteroids are easier to setup and mature in 4–7 days (Figure 3).

*Salmonella* infections induce intestinal inflammatory responses involving neutrophil infiltrations and the production of pro-inflammatory cytokines. It has been shown that cytokine secretions were enhanced in monolayer and 3-D organotypic models of human colonic epithelium (HT-29) as well as in the C57BL/6 mouse model upon *Salmonella* infection (Höner Zu Bentrup et al., 2006; Ren et al., 2009). Furthermore, patients infected by *Salmonella* display gamma interferon (IFN-γ) induction together with elevated tumor necrosis factor alpha (TNF-α) (Gal-Mor et al., 2012). Using crypt-derived mouse enteroids, Zhang et al. were able to reproduce the *Salmonella*-induced inflammatory responses (Zhang Y. G. et al., 2014). Furthermore, *Salmonella* infection induced the activation of nuclear factor kappa-light-chain-enhancer of activated B cells (NF-κB) signaling pathway in the mouse enteroids accompanied by the expression of inflammatory cytokines including TNF-α and IFN-γ (Zhang K. et al., 2014). This is in line with results from *Salmonella* infection of iHOs where genes encoding proinflammatory cytokines were upregulated (Forbester et al., 2015). Moreover, *Salmonella* infection significantly decreased the expression of intestinal stem cell markers, Lgr5 and Bmi 1 (Forbester et al., 2015). The significance and mechanism behind this decrease require further investigation using the enteroids model.

Invasion of the intestinal epithelium is an essential step for *Salmonella* virulence (Galán and Curtiss, 1989; Galan and Zhou, 2000). A study using the crypt-derived mouse enteroids (6 days after passage) showed that *Salmonella* quickly attached and invaded the enteroids (Zhang Y. G. et al., 2014) accompanied by the typical morphologic changes of the host cells during *Salmonella* invasion as well as the disruption of epithelial tight junctions (Finlay et al., 1991; Galan and Zhou, 2000; Zhang Y. G. et al., 2014). It is further shown that wild type *Salmonella* strains microinjected into the lumen of iHOs are able to invade the epithelial layer, and continue to traffic inside the *Salmonella*-containing vacuoles. In contrast, a *Salmonella invA* mutant, defective in the *Salmonella* pathogenicity island 1 invasion apparatus, was less capable of invading the iHO epithelium (Forbester et al., 2015). Furthermore, mouse enteroids were utilized to study the survival and replication of *Salmonella* and found that naturally secreted α-defensins by Paneth cells in the lumen suppressed the growth of *Salmonella* (Wilson et al., 2015). This finding is in agreement with data obtained when using *ex vivo* intestinal crypts and villus (Ayabe et al., 2000).

Chronic *Salmonella* Typhi infections are one of the reported risk factors for Gallbladder carcinoma (GBC) (Wistuba and Gazdar, 2004). A recent study showed that *Salmonella* infections induced malignant transformation in murine gallbladder organoids (Scanu et al., 2015). Interestingly, Scanu et al. found that *Salmonella*-mediated activation of mitogen-activated protein kinase (MAPK) and protein kinase B (PKB or AKT) pathways is responsible for the transformation. Importantly, the result from murine gallbladder organoids is in agreement with observations in GBC patients (Scanu et al., 2015). Collectively, both iHOs and enteroids have been successfully used for dissecting *Salmonella* pathogenesis. Enteroids may provide a promising experimental platform to investigate *Salmonella* infections for antimicrobial drug screening and personalized medicine.

## Lessons learned from studies of other enteropathogens

Conventional 2-D monolayer cultures have greatly aided the advancement of our understanding of host-pathogen interactions despite their limitations in single cell type and being tumor-derived (Duell et al., 2011). It is known that different pathogens may preferentially infect a subset of cell types and could exploit different host molecules to promote their infections. In addition, many biological processes that drive immune responses against pathogens are difficult if not impossible to mimic using just monolayer cell line cultures (Duell et al., 2011). Many infectious agents have been shown to infect enteroids (Table 2), including parasites (Gerbe et al., 2016), rotavirus (Yin et al., 2015), norovirus (Ettayebi et al., 2016), Enterohemorrhagic *Escherichia coli* (In et al., 2016), and *Salmonella* (Zhang Y. G. et al., 2014; Scanu et al., 2015; Wilson et al., 2015).

**Table 2:** Various intestinal physiology and disease processes have been modeled by organoids and enteroids.

	**Year**	**Author**	**Modeling disease**
Non-infectious diseases	2013	Dekkers et al.	Cystic fibrosis caused by mutations of CFTR genes (Dekkers et al., 2013)
	2015	Matano et al.	Colorectal cancer (Matano et al., 2015)
	2016	Zachos et al.	Transport of electrolytes and intestinal fluid (Zachos et al., 2016)
	2017	Noben et al.	Inflammatory bowel disease including ulcerative colitis and Crohn's disease (Noben et al., 2017)
	2017	Zou et al.	Evaluation of the effectiveness of personalized medicine on chemotherapy drugs (Zou et al., 2017)
Infectious diseases	2014	Foulke-Abel et al.	Rotavirus (Foulke-Abel et al., 2014)
	2014	Zhang et al.	*Salmonella* (Zhang Y. G. et al., 2014)
	2015	Bartfelt et al.	*Helicobacter pylori (H. pylori)* (Bartfeld and Clevers, 2015)
	2015	Forbester et al.	*Salmonella enterica* serovar Typhimurium (Forbester et al., 2015)
	2015	Wilson et al.	*Salmonella enterica* serovar Typhimurium (Wilson et al., 2015)
	2015	Leslie et al.	*Clostridia difficile (C. difficile)* (Leslie et al., 2015)
	2015	Yin et al.	Rotavirus (Yin et al., 2015)
	2016	Ettayebi et al.	Norovirus (Ettayebi et al., 2016)
	2016	Gerbe et al.	Parasites (Gerbe et al., 2016)
	2016	In et al.	Enterohemorrhagic *Escherichia coli* (In et al., 2016)
	2016	Yin et al.	Rotavirus (Yin et al., 2016)
	2017	Karve et al.	*E. coli* O157:H7 (Karve et al., 2017)
	2017	Wilson et al.	Mouse adenovirus 2 (MAdV-2) (Wilson et al., 2017)
	2017	Yin et al.	Rotavirus (Yin et al., 2017)

Rotavirus is the leading cause of gastroenteritis and diarrhea in worldwide. Rotavirus is known to target the human intestinal epithelial cells and was shown to infect human enteroids (Foulke-Abel et al., 2014; Yin et al., 2015). This significant advance overcame the fact that human rotavirus replicates poorly in transformed cell lines. In addition, many animal models have limited use due to host range restrictions of rotavirus. It was shown that rotavirus infections led physiological lumenal expansion, a hallmark of rotavirus-induced diarrhea (Saxena et al., 2015). Laboratory adapted rotavirus strains and patient derived isolates were able to infect both mouse and human enteroids. Interestingly, human enteroids were more susceptible to human rotavirus infections than mouse enteroids (Yin et al., 2015). Moreover, patient derived rotavirus showed different infectivity in response to commonly used antiviral drugs including ribavirin and IFNα from that of the laboratory adapted rotavirus when infecting human enteroids (Yin et al., 2015). Rotavirus also induced less pronounced expression of genes involved in innate immune responses (interferon stimulated genes, ISGs) in enteroids than that in Caco2 cells (Yin et al., 2015). Consistent with clinical patient data, the antiviral effect of IFNα was less in enteroids as compared to that in Caco2 cells (Yin et al., 2015). Recently, enteroids developed from transgenic mice have been successfully used to characterize NOD-like receptor (NLR) Nlrp9b inflammasome-mediated rotavirus restriction in intestinal epithelial cells in mice (Zhu et al., 2017). Therefore, enteroids derived from a variety of transgenic mice highlight their potential to contribute to the understanding of molecular mechanisms during intestinal epithelial cell infections.

Norovirus is another enteric virus causes severe gastroenteritis in both infants and adults (Ettayebi et al., 2016). It is known that cultured cell lines do not support the replication of norovirus (Papafragkou et al., 2014). 3-D intestinal model derived from INT-407 cells and Caco2 cells also failed to facilitate norovirus replication (Papafragkou et al., 2014). In contrast, enteroids were shown to support norovirus infection and clear cytopathic effects (CPE) and viral particles were observed upon norovirus infection. The viral replication was even more pronounced by adding bile to the growth media. Human enteroids were also recently reported to be used to examine roles of secreted alpha-defensins during infection by mouse adenovirus 2 (Wilson et al., 2017).

In addition to viral studies, enteroids have been used successfully to explore bacterial pathogeneses besides *Salmonella*. Enterohemorrhagic *Escherichia coli* (EHEC) cause foodborne diseases in both developing and industrialized countries (In et al., 2016). Recently, human enteroids with the addition of human neutrophils were established to study *E. coli* O157:H7 infections (Karve et al., 2017). In the lumen of enteroids, pathogenic O157:H7 replicated rapidly while commensal *E. coli* did not (Karve et al., 2017). Interestingly, O157:H7 infections promoted the recruitment of human neutrophils (Karve et al., 2017). Furthermore, enteroids provided a unique model to study the interactions between the gut microbiota, enteric pathogens, and the host intestinal environment (Nigro et al., 2016).

*Clostridium difficile* (*C. difficile*), a Gram-positive obligate anaerobic bacteria ubiquitous in nature, infects the human colon in 2–5% of the adult population. Imbalance of the normal gut flora could increase the chances of *Clostridium* infection. The bacteria may produce diarrhea and inflammation in infected patients via the well characterized enterotoxin (*C. difficile* toxin A) and cytotoxin (*C. difficile* toxin B). IHIOs derived from human pluripotent stem cells were used to study *C. difficile* and the contribution of toxins. It was shown that the toxins played a major role in the colonization and disruption of the iHIO epithelium, and in the loss of the paracellular barrier function (Leslie et al., 2015).

*Helicobacter pylori* (*H. pylori*) colonization of the human stomach has been associated with chronic gastritis, ulceration, and adenocarcinoma. To study the pathogenesis of *H. pylori* infection, human gastric organoids were generated from surgical samples of human gastric corpus. The gastric organoids displayed the typical characteristics of their corresponding tissues, based on their histology, expression of markers, and euploidy (Bartfeld et al., 2015). This system has the potential to be used to study other gastric pathologies in addition to *H. pylori* infection.

One exciting potential use of patient-derived enteroids is to evaluate the effectiveness of personalized medicine, such as precision chemotherapy for cancer patients. Roy et al. successfully evaluated the effects of Mitomycin-C, 5-Fluorouracil (5-FU), Irinotecan, Oxaliplatin, Doxorubicin, and Paclitaxel on peritoneal metastases using enteroids (Roy et al., 2017). Enteroids have also been used to model various intestinal physiology and disease processes (Table 2). Human enteroids have been used to study the transport of electrolytes and intestinal fluid using luminal dilatation assay (Zachos et al., 2016). Enteroids have also contributed to the understanding of cystic fibrosis caused by mutations in the cystic fibrosis transmembrane conductance regulator (CFTR) using a swelling assay (Dekkers et al., 2013). Moreover, enteroids have been used to model colorectal cancer and inflammatory bowel disease including ulcerative colitis and Crohn's disease (Matano et al., 2015; Young and Reed, 2016; Noben et al., 2017). Finally, the potential transplantation of enteroids into patients with intestinal failure (IF), a life-threatening condition, further expanded the possibility of using enteroids to treat patients (Hong et al., 2017). Taken together, enteroids are emerging as robust infection models for study both viral and bacterial infections and may play a key role in the development of personalized medicine to aid the treatment of human infections and diseases.

## Summary and conclusions

Salmonellosis remains a major public health concern globally. The availability of various infection models has helped to identify bacterial virulence factors responsible for causing the diseases. Studies utilizing these infection models have advanced our understanding of how pathogens deploy their virulence factors to modulate host cell functions during infection. Classical infection models include various 2-D cultures of immortalized cells and animal models. Recent advances in stem cell research have helped to establish organoids and enteroids as viable alternatives to many established infection models. We anticipate that organoids and enteroid infection models will play a key role in advancing out understanding in antimicrobial drug screening, personalized medicine, virulence mechanisms and pathogen-host interactions.

## Author contributions

All authors listed have made a substantial, direct and intellectual contribution to the work, and approved it for publication.

### Conflict of interest statement

The authors declare that the research was conducted in the absence of any commercial or financial relationships that could be construed as a potential conflict of interest.

## References

[B1] Ayabe T. Satchell D. P. Wilson C. L. Parks W. C. Selsted M. E. Ouellette A. J. (2000). Secretion of microbicidal alpha-defensins by intestinal Paneth cells in response to bacteria. Nat. Immunol. 1, 113–118. 10.1038/77783 11248802

[B2a] Barrila J. Radtke A. L. Crabbe A. Sarker S. F. Herbst-Kralovetz M. M. Ott C. M.. (2010). Organotypic 3D cell culture models: using the rotating wall vessel to study host-pathogen interactions. Nat. Rev. Microbiol. 8, 791–801. 10.1038/nrmicro2423 20948552

[B2] Bartfeld S. Bayram T. Van De Wetering M. Huch M. Begthel H. Kujala P.. (2015). *In vitro* expansion of human gastric epithelial stem cells and their responses to bacterial infection. Gastroenterology 148, 126–136 e126. 10.1053/j.gastro.2014.09.042 25307862 PMC4274199

[B4a] Bartfeld S. Clevers H. (2015). Organoids as model for infectious diseases: culture of human and murine stomach organoids and microinjection of *Helicobacter pylori*. J. Vis Exp. 10.3791/53359. [Epub ahead of print]. 26650279 PMC4692704

[B3] Barthel M. Hapfelmeier S. Quintanilla-Martínez L. Kremer M. Rohde M. Hogardt M.. (2003). Pretreatment of mice with streptomycin provides a *Salmonella enterica* Serovar Typhimurium Colitis Model that allows analysis of both pathogen and host. Infect. Immun. 71, 2839–2858. 10.1128/IAI.71.5.2839-2858.2003 12704158 PMC153285

[B4] Blanc-Potard A. B. Groisman E. A. (1997). The Salmonella selC locus contains a pathogenicity island mediating intramacrophage survival. EMBO J. 16, 5376–5385. 10.1093/emboj/16.17.5376 9311997 PMC1170169

[B5] Boyle E. C. Bishop J. L. Grassl G. A. Finlay B. B. (2007). Salmonella: from pathogenesis to therapeutics. J. Bacteriol. 189, 1489–1495. 10.1128/JB.01730-06 17189373 PMC1855715

[B8a] Boyle E. C. Dombrowsky H. Sarau J. Braun J. Aepfelbacher M. Lautenschläger I.. (2015). Ex vivo perfusion of the isolated rat small intestine as a novel model of Salmonella enteritis. Am. J. Physiol. Gastrointest. Liver Physiol. 310, G55–G63. 10.1152/ajpgi.00444.2014 26564721

[B6] Brenner F. W. Villar R. G. Angulo F. J. Tauxe R. Swaminathan B. (2000). Salmonella nomenclature. J. Clin. Microbiol. 38, 2465–2467. 10878026 10.1128/jcm.38.7.2465-2467.2000 PMC86943

[B7] Buchmeier N. A. Heffron F. (1989). Intracellular survival of wild-type Salmonella typhimurium and macrophage-sensitive mutants in diverse populations of macrophages. Infect. Immun. 57, 1–7. 2642463 10.1128/iai.57.1.1-7.1989 PMC313031

[B8] Clark M. A. Jepson M. A. Simmons N. L. Hirst B. H. (1994). Preferential interaction of Salmonella typhimurium with mouse Peyer's patch M cells. Res. Microbiol. 145, 543–552. 10.1016/0923-2508(94)90031-0 7855440

[B9] Clark M. A. Reed K. A. Lodge J. Stephen J. Hirst B. H. Jepson M. A. (1996). Invasion of murine intestinal M cells by Salmonella typhimurium inv mutants severely deficient for invasion of cultured cells. Infect. Immun. 64, 4363–4368. 10.1128/iai.64.10.4363-4368.1996 PMC174381 8926113

[B10] Coburn B. Grassl G. A. Finlay B. B. (2007). Salmonella, the host and disease: a brief review. Immunol. Cell Biol. 85, 112–118. 10.1038/sj.icb.7100007 17146467

[B11] Crawford R. W. Keestra A. M. Winter S. E. Xavier M. N. Tsolis R. M. Tolstikov V.. (2012). Very long O-antigen chains enhance fitness during Salmonella-induced colitis by increasing bile resistance. PLoS Pathog. 8:e1002918. 10.1371/journal.ppat.1002918 23028318 PMC3447750

[B12] Crump J. A. Sjölund-Karlsson M. Gordon M. A. Parry C. M. (2015). Epidemiology, clinical presentation, laboratory diagnosis, antimicrobial resistance, and antimicrobial management of invasive Salmonella infections. Clin. Microbiol. Rev. 28, 901–937. 10.1128/CMR.00002-15 26180063 PMC4503790

[B13] de Lau W. Kujala P. Schneeberger K. Middendorp S. Li V. S. Barker N.. (2012). Peyer's patch M cells derived from Lgr5(+) stem cells require SpiB and are induced by RankL in cultured “miniguts”. Mol. Cell. Biol. 32, 3639–3647. 10.1128/MCB.00434-12 22778137 PMC3430189

[B14] Dekkers J. F. Wiegerinck C. L. De Jonge H. R. Bronsveld I. Janssens H. M. De Winter-De Groot K. M.. (2013). A functional CFTR assay using primary cystic fibrosis intestinal organoids. Nat. Med. 19, 939–945. 10.1038/nm.3201 23727931

[B18a] Dostal A. Gagnon M. Chassard C. Zimmermann M. B. O'mahony L. Lacroix C. (2014). Salmonella adhesion, invasion and cellular immune responses are differentially affected by iron concentrations in a combined *in vitro* gut fermentation-cell model. PLoS ONE 9:e93549. 10.1371/journal.pone.0093549 24676135 PMC3968171

[B15] Driehuis E. Clevers H. (2017). CRISPR/Cas 9 genome editing and its applications in organoids. Am. J. Physiol. Gastrointest. Liver Physiol. 312, G257–G265. 10.1152/ajpgi.00410.2016 28126704

[B16] Duell B. L. Cripps A. W. Schembri M. A. Ulett G. C. (2011). Epithelial cell coculture models for studying infectious diseases: benefits and limitations. J. Biomed. Biotechnol. 2011:852419. 10.1155/2011/852419 22007147 PMC3189631

[B17] Dutta D. Heo I. Clevers H. (2017). Disease modeling in stem cell-derived 3D organoid systems. Trends Mol. Med. 23, 393–410. 10.1016/j.molmed.2017.02.007 28341301

[B18] Eng S.-K. Pusparajah P. Ab Mutalib N.-S. Ser H.-L. Chan K.-G. Lee L.-H. (2015). Salmonella: a review on pathogenesis, epidemiology and antibiotic resistance. Front. Life Sci. 8, 284–293. 10.1080/21553769.2015.1051243

[B19] Ettayebi K. Crawford S. E. Murakami K. Broughman J. R. Karandikar U. Tenge V. R.. (2016). Replication of human noroviruses in stem cell-derived human enteroids. Science 353, 1387–1393. 10.1126/science.aaf5211 27562956 PMC5305121

[B20] Fang S. B. Huang C. J. Huang C. H. Wang K. C. Chang N. W. Pan H. Y.. (2017). speG Is required for intracellular replication of Salmonella in various human cells and affects its polyamine metabolism and global transcriptomes. Front. Microbiol. 8:2245. 10.3389/fmicb.2017.02245 29187844 PMC5694781

[B21] Fierer J. Guiney D. G. (2001). Diverse virulence traits underlying different clinical outcomes of Salmonella infection. J. Clin. Invest. 107, 775–780. 10.1172/JCI12561 11285291 PMC199580

[B22] Finlay B. B. Brumell J. H. (2000). Salmonella interactions with host cells: *in vitro* to *in vivo*. Philos. Trans. R. Soc. Lond. B. Biol. Sci. 355, 623–631. 10.1098/rstb.2000.0603 10874735 PMC1692772

[B23] Finlay B. B. Ruschkowski S. Dedhar S. (1991). Cytoskeletal rearrangements accompanying Salmonella entry into epithelial-cells. J. Cell Sci. 99, 283–293. 1909337 10.1242/jcs.99.2.283

[B24] Firoz Mian M. Pek E. A. Chenoweth M. J. Ashkar A. A. (2011). Humanized mice are susceptible to Salmonella typhi infection. Cell. Mol. Immunol. 8, 83–87. 10.1038/cmi.2010.52 21200387 PMC4002987

[B25] Forbester J. L. Goulding D. Vallier L. Hannan N. Hale C. Pickard D.. (2015). Interaction of *Salmonella enterica* Serovar Typhimurium with intestinal organoids derived from human induced pluripotent stem cells. Infect. Immun. 83, 2926–2934. 10.1128/IAI.00161-15 25964470 PMC4468523

[B26] Foster J. W. (1995). Low pH adaptation and the acid tolerance response of Salmonella typhimurium. Crit. Rev. Microbiol. 21, 215–237. 10.3109/10408419509113541 8688153

[B27] Foulke-Abel J. In J. Kovbasnjuk O. Zachos N. C. Ettayebi K. Blutt S. E.. (2014). Human enteroids as an *ex-vivo* model of host-pathogen interactions in the gastrointestinal tract. Exp. Biol. Med. (Maywood) 239, 1124–1134. 10.1177/1535370214529398 24719375 PMC4380516

[B28] Frost A. J. Bland A. P. Wallis T. S. (1997). The early dynamic response of the calf ileal epithelium to Salmonella typhimurium. Vet. Pathol. 34, 369–386. 10.1177/030098589703400501 9381648

[B29] Galán J. E. (1998). Interactions of Salmonella with host cells: encounters of the closest kind. Proc. Natl. Acad. Sci. U. S. A. 95, 14006–14008. 10.1073/pnas.95.24.14006 9826642 PMC33922

[B30] Galán J. E. (1999). Interaction of Salmonella with host cells through the centisome 63 type III secretion system. Curr. Opin. Microbiol. 2, 46–50. 10.1016/S1369-5274(99)80008-3 10047557

[B31] Galán J. E. Curtiss R. III. (1989). Cloning and molecular characterization of genes whose products allow Salmonella typhimurium to penetrate tissue culture cells. Proc. Natl. Acad. Sci. U.S.A. 86, 6383–6387. 10.1073/pnas.86.16.6383 2548211 PMC297844

[B32] Galan J. E. Zhou D. (2000). Striking a balance: modulation of the actin cytoskeleton by Salmonella. Proc. Natl. Acad. Sci. U.S.A. 97, 8754–8761. 10.1073/pnas.97.16.8754 10922031 PMC34008

[B33] Gal-Mor O. Suez J. Elhadad D. Porwollik S. Leshem E. Valinsky L.. (2012). Molecular and cellular characterization of a *Salmonella enterica* serovar Paratyphi a outbreak strain and the human immune response to infection. Clin. Vaccine Immunol. 19, 146–156. 10.1128/CVI.05468-11 22190395 PMC3272918

[B34] Gerbe F. Sidot E. Smyth D. J. Ohmoto M. Matsumoto I. Dardalhon V.. (2016). Intestinal epithelial tuft cells initiate type 2 mucosal immunity to helminth parasites. Nature 529, 226–230. 10.1038/nature16527 26762460 PMC7614903

[B35] Giannella R. A. Formal S. B. Dammin G. J. Collins H. (1973). Pathogenesis of salmonellosis. Studies of fluid secretion, mucosal invasion, and morphologic reaction in the rabbit ileum. J. Clin. Invest. 52, 441–453. 10.1172/JCI107201 4630603 PMC302274

[B36] Giannella R. A. Rout W. R. Formal S. B. (1977). Effect of indomethacin on intestinal water transport in salmonella-infected rhesus monkeys. Infect. Immun. 17, 136–139. 407160 10.1128/iai.17.1.136-139.1977 PMC421093

[B37] Gordon M. A. (2011). Invasive Non-typhoidal Salmonella Disease–epidemiology, pathogenesis and diagnosis. Curr. Opin. Infect. Dis. 24:484 10.1097/QCO.0b013e32834a9980 21844803 PMC3277940

[B38] Griffin A. J. McSorley S. J. (2011). Development of protective immunity to Salmonella, a mucosal pathogen with a systemic agenda. Mucosal Immunol. 4, 371–382. 10.1038/mi.2011.2 21307847 PMC4084725

[B39] Guiney D. G. Fang F. C. Krause M. Libby S. (1994). Plasmid-mediated virulence genes in non-typhoid Salmonella serovars. FEMS Microbiol. Lett. 124, 1–9. 10.1111/j.1574-6968.1994.tb07253.x 8001760

[B40] Gulig P. A. (1990). Virulence plasmids of Salmonella typhimurium and other salmonellae. Microb. Pathog. 8, 3–11. 10.1016/0882-4010(90)90003-9 2185396

[B41] Haque A. Bowe F. Fitzhenry R. J. Frankel G. Thomson M. Heuschkel R.. (2004). Early interactions of *Salmonella enterica* serovar typhimurium with human small intestinal epithelial explants. Gut 53, 1424–1430. 10.1136/gut.2003.037382 15361488 PMC1774215

[B42] Hensel M. Shea J. E. Raupach B. Monack D. Falkow S. Gleeson C.. (1997). Functional analysis of ssaJ and the ssaK/U operon, 13 genes encoding components of the type III secretion apparatus of Salmonella Pathogenicity Island 2. Mol. Microbiol. 24, 155–167. 10.1046/j.1365-2958.1997.3271699.x 9140973

[B43] Hensel M. Shea J. E. Waterman S. R. Mundy R. Nikolaus T. Banks G.. (1998). Genes encoding putative effector proteins of the type III secretion system of Salmonella pathogenicity island 2 are required for bacterial virulence and proliferation in macrophages. Mol. Microbiol. 30, 163–174. 10.1046/j.1365-2958.1998.01047.x 9786193

[B44] Höner Zu Bentrup K. Ramamurthy R. Ott C. M. Emami K. Nelman-Gonzalez M. Wilson J. W.. (2006). Three-dimensional organotypic models of human colonic epithelium to study the early stages of enteric salmonellosis. Microbes Infect. 8, 1813–1825. 10.1016/j.micinf.2006.02.020 16730210

[B45] Hong S. N. Dunn J. C. Stelzner M. Martín M. G. (2017). Concise review: the potential use of intestinal stem cells to treat patients with intestinal failure. Stem Cells Transl. Med. 6, 666–676. 10.5966/sctm.2016-0153 28191783 PMC5442796

[B46] Hurley B. P. McCormick B. A. (2003). Translating tissue culture results into animal models: the case of Salmonella typhimurium. Trends Microbiol. 11, 562–569. 10.1016/j.tim.2003.10.002 14659688

[B47] Hurley D. Mccusker M. P. Fanning S. Martins M. (2014). Salmonella-host interactions - modulation of the host innate immune system. Front Immunol 5:481. 10.3389/fimmu.2014.00481 25339955 PMC4188169

[B48] In J. Foulke-Abel J. Zachos N. C. Hansen A. M. Kaper J. B. Bernstein H. D.. (2016). Enterohemorrhagic *Escherichia coli* reduce mucus and intermicrovillar bridges in human stem cell-derived colonoids. Cell. Mol. Gastroenterol. Hepatol. 2, 48–62.e43. 10.1016/j.jcmgh.2015.10.001 26855967 PMC4740923

[B49] Itri F. Monti D. M. Chino M. Vinciguerra R. Altucci C. Lombardi A.. (2017). Identification of novel direct protein-protein interactions by irradiating living cells with femtosecond UV laser pulses. Biochem. Biophys. Res. Commun. 492, 67–73. 10.1016/j.bbrc.2017.08.037 28807828

[B50] Jones B. D. Ghori N. Falkow S. (1994). Salmonella typhimurium initiates murine infection by penetrating and destroying the specialized epithelial M cells of the Peyer's patches. J. Exp. Med. 180, 15–23. 10.1084/jem.180.1.15 8006579 PMC2191576

[B51] Karve S. S. Pradhan S. Ward D. V. Weiss A. A. (2017). Intestinal organoids model human responses to infection by commensal and Shiga toxin producing *Escherichia coli*. PLoS ONE 12:e0178966. 10.1371/journal.pone.0178966 28614372 PMC5470682

[B52] Kinsey M. D. Dammin G. J. Formal S. B. Giannella R. A. (1976). The role of altered intestinal permeability in the pathogenesis of salmonella diarrhea in the rhesus monkey. Gastroenterology 71, 429–434. 820589

[B53] Kubori T. Matsushima Y. Nakamura D. Uralil J. Lara-Tejero M. Sukhan A.. (1998). Supramolecular structure of the Salmonella typhimurium type III protein secretion system. Science 280, 602–605. 10.1126/science.280.5363.602 9554854

[B54] Kumar G. Pratap C. B. Mishra O. P. Kumar K. Nath G. (2012). Use of urine with nested PCR targeting the flagellin gene (fliC) for diagnosis of typhoid fever. J. Clin. Microbiol. 50, 1964–1967. 10.1128/JCM.00031-12 22493333 PMC3372149

[B55] Le Blay G. Rytka J. Zihler A. Lacroix C. (2009). New *in vitro* colonic fermentation model for Salmonella infection in the child gut. FEMS Microbiol. Ecol. 67, 198–207. 10.1111/j.1574-6941.2008.00625.x 19087202

[B56] Legrand N. Ploss A. Balling R. Becker P. D. Borsotti C. Brezillon N.. (2009). Humanized mice for modeling human infectious disease: challenges, progress, and outlook. Cell Host Microbe 6, 5–9. 10.1016/j.chom.2009.06.006 19616761 PMC7038630

[B57] Leslie J. L. Huang S. Opp J. S. Nagy M. S. Kobayashi M. Young V. B.. (2015). Persistence and toxin production by *Clostridium difficile* within human intestinal organoids result in disruption of epithelial paracellular barrier function. Infect. Immun. 83, 138–145. 10.1128/IAI.02561-14 25312952 PMC4288864

[B58] Lindquist B. L. Lebenthal E. Lee P. C. Stinson M. W. Merrick J. M. (1987). Adherence of Salmonella typhimurium to small-intestinal enterocytes of the rat. Infect. Immun. 55, 3044–3050. 2890583 10.1128/iai.55.12.3044-3050.1987 PMC260026

[B59] Lopez C. A. Rivera-Chávez F. Byndloss M. X. Báumler A. J. (2015). The Periplasmic Nitrate Reductase NapABC supports luminal growth of *Salmonella enterica* Serovar Typhimurium during Colitis. Infect. Immun. 83, 3470–3478. 10.1128/IAI.00351-15 26099579 PMC4534643

[B60] Martinez-Argudo I. Jepson M. A. (2008). Salmonella translocates across an *in vitro* M cell model independently of SPI-1 and SPI-2. Microbiology 154, 3887–3894. 10.1099/mic.0.2008/021162-0 19047755

[B61] Matano M. Date S. Shimokawa M. Takano A. Fujii M. Ohta Y.. (2015). Modeling colorectal cancer using CRISPR-Cas9-mediated engineering of human intestinal organoids. Nat. Med. 21, 256–262. 10.1038/nm.3802 25706875

[B66a] Mathur R. Oh H. Zhang D. Park S. G. Seo J. Koblansky A.. (2012). A mouse model of salmonella typhi infection. Cell 151, 590–602. 10.1016/j.cell.2012.08.042 23101627 PMC3500584

[B62] McGovern V. J. Slavutin L. J. (1979). Pathology of salmonella colitis. Am. J. Surg. Pathol. 3, 483–490. 10.1097/00000478-197912000-00001 534385

[B63] Miyoshi H. Stappenbeck T. S. (2013). *In vitro* expansion and genetic modification of gastrointestinal stem cells in spheroid culture. Nat. Protocols 8, 2471–2482. 10.1038/nprot.2013.153 24232249 PMC3969856

[B64] Moulder J. W. (1985). Comparative biology of intracellular parasitism. Microbiol. Rev. 49, 298–337. 3900672 10.1128/mr.49.3.298-337.1985 PMC373037

[B65] Newburg D. S. Ko J. S. Leone S. Nanthakumar N. N. (2016). Human Milk Oligosaccharides and Synthetic Galactosyloligosaccharides Contain 3′-, 4-, and 6′-Galactosyllactose and Attenuate Inflammation in Human T84, NCM-460, and H4 Cells and Intestinal Tissue Ex Vivo. J. Nutr. 146, 358–367. 10.3945/jn.115.220749 26701795 PMC4725434

[B66] Nickerson C. A. Goodwin T. J. Terlonge J. Ott C. M. Buchanan K. L. Uicker W. C.. (2001). Three-dimensional tissue assemblies: novel models for the study of *Salmonella enterica* serovar Typhimurium pathogenesis. Infect. Immun. 69, 7106–7120. 10.1128/IAI.69.11.7106-7120.2001 11598087 PMC100098

[B67] Nietfeld J. C. Tyler D. E. Harrison L. R. Cole J. R. Latimer K. S. Crowell W. A. (1992). Invasion of enterocytes in cultured porcine small intestinal mucosal explants by Salmonella choleraesuis. Am. J. Vet. Res. 53, 1493–1499. 1416346

[B68] Nigro G. Hanson M. Fevre C. Lecuit M. Sansonetti P. J. (2016). Intestinal organoids as a novel tool to study microbes-epithelium interactions. Methods Mol. Biol. [Epub ahead of print]. 10.1007/7651_2016_12 27628134

[B69] Noben M. Verstockt B. De Bruyn M. Hendriks N. Van Assche G. Vermeire S.. (2017). Epithelial organoid cultures from patients with ulcerative colitis and Crohn's disease: a truly long-term model to study the molecular basis for inflammatory bowel disease? Gut 66, 2193–2195. 10.1136/gutjnl-2016-313667 28159838

[B70] Norris F. A. Wilson M. P. Wallis T. S. Galyov E. E. Majerus P. W. (1998). SopB, a protein required for virulence of Salmonella dublin, is an inositol phosphate phosphatase. Proc. Natl. Acad. Sci. U.S.A. 95, 14057–14059. 10.1073/pnas.95.24.14057 9826652 PMC24325

[B71] Ochman H. Soncini F. C. Solomon F. Groisman E. A. (1996). Identification of a pathogenicity island required for Salmonella survival in host cells. Proc. Natl. Acad. Sci. U.S.A. 93, 7800–7804. 10.1073/pnas.93.15.7800 8755556 PMC38828

[B72] Özkaya H. Akcan A. B. Aydemir G. AydinÖz S. Razia Y. Gammon S. T.. (2012). Salmonella typhimurium infections in BALB/c mice: a comparison of tissue bioluminescence, tissue cultures and mice clinical scores. New Microbiol. 35, 53–59. 22378553

[B73] Papafragkou E. Hewitt J. Park G. W. Greening G. Vinjé J. (2014). Challenges of culturing human norovirus in three-dimensional organoid intestinal cell culture models. PLoS ONE 8:e63485. 10.1371/journal.pone.0063485 23755105 PMC3670855

[B74] Popoff M. Y. Bockemühl J. Gheesling L. L. (2003). Supplement 2001 (no. 45) to the Kauffmann-White scheme. Res. Microbiol. 154, 173–174. 10.1016/S0923-2508(03)00025-1 12706505

[B75] Ren Z. Gay R. Thomas A. Pae M. Wu D. Logsdon L.. (2009). Effect of age on susceptibility to Salmonella Typhimurium infection in C57BL/6 mice. J. Med. Microbiol. 58, 1559–1567. 10.1099/jmm.0.013250-0 19729455 PMC2783761

[B76] Rivera-Chávez F. Báumler A. J. (2015). The pyromaniac inside you: Salmonella metabolism in the host gut. Annu. Rev. Microbiol. 69, 31–48. 10.1146/annurev-micro-091014-104108 26002180

[B77] Rivera-Chávez F. Winter S. E. Lopez C. A. Xavier M. N. Winter M. G. Nuccio S. P.. (2013). Salmonella uses energy taxis to benefit from intestinal inflammation. PLoS Pathog. 9:e1003267. 10.1371/journal.ppat.1003267 23637594 PMC3630101

[B78] Rivera-Chávez F. Zhang L. F. Faber F. Lopez C. A. Byndloss M. X. Olsan E. E.. (2016). Depletion of butyrate-producing clostridia from the gut microbiota drives an aerobic luminal expansion of Salmonella. Cell Host Microbe 19, 443–454. 10.1016/j.chom.2016.03.004 27078066 PMC4832419

[B79] Roy P. Canet-Jourdan C. Annereau M. Zajac O. Gelli M. Broutin S.. (2017). Organoids as preclinical models to improve intraperitoneal chemotherapy effectiveness for colorectal cancer patients with peritoneal metastases: preclinical models to improve HIPEC. Int. J. Pharm. 531, 143–152. 10.1016/j.ijpharm.2017.07.084 28803938

[B80] Samuel J. L. O'boyle D. A. Mathers W. J. Frost A. J. (1980). Distribution of Salmonella in the carcases of normal cattle at slaughter. Res. Vet. Sci. 28, 368–372. 7414091

[B81] Sansonetti P. J. Phalipon A. (1999). M cells as ports of entry for enteroinvasive pathogens: mechanisms of interaction, consequences for the disease process. Semin. Immunol. 11, 193–203. 10.1006/smim.1999.0175 10381865

[B82] Santos R. L. Zhang S. Tsolis R. M. Kingsley R. A. Adams L. G. Bäumler A. J. (2001). Animal models of Salmonella infections: enteritis versus typhoid fever. Microbes Infect. 3, 1335–1344. 10.1016/S1286-4579(01)01495-2 11755423

[B83] Sato T. Clevers H. (2013). Growing self-organizing mini-guts from a single intestinal stem cell: mechanism and applications. Science 340, 1190–1194. 10.1126/science.1234852 23744940

[B84] Sato T. Stange D. E. Ferrante M. Vries R. G. Van Es J. H. Van Den Brink S.. (2011). Long-term expansion of epithelial organoids from human colon, adenoma, adenocarcinoma, and Barrett's epithelium. Gastroenterology 141, 1762–1772. 10.1053/j.gastro.2011.07.050 21889923

[B85] Sato T. Vries R. G. Snippert H. J. Van De Wetering M. Barker N. Stange D. E.. (2009). Single Lgr5 stem cells build crypt-villus structures *in vitro* without a mesenchymal niche. Nature 459, 262–265. 10.1038/nature07935 19329995

[B86] Saxena K. Blutt S. E. Ettayebi K. Zeng X. L. Broughman J. R. Crawford S. E.. (2015). Human intestinal enteroids: a new model to study human rotavirus infection, host restriction, and pathophysiology. J. Virol. 90, 43–56. 10.1128/JVI.01930-15 26446608 PMC4702582

[B87] Scanu T. Spaapen R. M. Bakker J. M. Pratap C. B. Wu L. E. Hofland I.. (2015). Salmonella manipulation of host signaling pathways provokes cellular transformation associated with gallbladder carcinoma. Cell Host Microbe 17, 763–774. 10.1016/j.chom.2015.05.002 26028364

[B88] Shea J. E. Beuzon C. R. Gleeson C. Mundy R. Holden D. W. (1999). Influence of the Salmonella typhimurium pathogenicity island 2 type III secretion system on bacterial growth in the mouse. Infect. Immun. 67, 213–219. 9864218 10.1128/iai.67.1.213-219.1999 PMC96299

[B89] Shea J. E. Hensel M. Gleeson C. Holden D. W. (1996). Identification of a virulence locus encoding a second type III secretion system in Salmonella typhimurium. Proc. Natl. Acad. Sci. U.S.A. 93, 2593–2597. 10.1073/pnas.93.6.2593 8637919 PMC39842

[B90] Stelzner M. Helmrath M. Dunn J. C. Henning S. J. Houchen C. W. Kuo C.. (2012). A nomenclature for intestinal *in vitro* cultures. Am. J. Physiol. Gastrointest. Liver Physiol. 302, G1359–G1363. 10.1152/ajpgi.00493.2011 22461030 PMC3378093

[B91] Takeuchi A. (1967). Electron microscope studies of experimental Salmonella infection. I. Penetration into the intestinal epithelium by Salmonella typhimurium. Am. J. Pathol. 50, 109–136. 5334433 PMC1965174

[B92] Tang H. J. Chen C. C. Zhang C. C. Cheng K. C. Chiang S. R. Chiu Y. H.. (2012). Use of Carbapenems against clinical, nontyphoid Salmonella isolates: results from *in vitro* and *in vivo* animal studies. Antimicrob. Agents Chemother. 56, 2916–2922. 10.1128/AAC.00110-12 22470122 PMC3370812

[B93] Tsilingiri K. Barbosa T. Penna G. Caprioli F. Sonzogni A. Viale G.. (2012). Probiotic and postbiotic activity in health and disease: comparison on a novel polarised *ex-vivo* organ culture model. Gut 61, 1007–1015. 10.1136/gutjnl-2011-300971 22301383

[B94] Tsolis R. M. Adams L. G. Ficht T. A. Bäumler A. J. (1999). Contribution of Salmonella typhimurium virulence factors to diarrheal disease in calves. Infect. Immun. 67, 4879–4885. 10456944 10.1128/iai.67.9.4879-4885.1999 PMC96822

[B95] Tsolis R. M. Adams L. G. Hantman M. J. Scherer C. A. Kimbrough T. Kingsley R. A. (2000). SspA is required for lethal *Salmonella enterica* serovar Typhimurium infections in calves but is not essential for diarrhea. Infect. Immun. 68, 3158–3163. 10.1128/IAI.68.6.3158-3163.2000 10816458 PMC97552

[B96] Van Lidth de Jeude J. F. Vermeulen J. L. M. Montenegro-Miranda P. S. Van Den Brink G. R. Heijmans J. (2015). A protocol for lentiviral transduction and downstream analysis of intestinal organoids. J. Vis. Exp. e52531. 10.3791/52531 25938265 PMC4541580

[B97] Vazquez-Torres A. Xu Y. Jones-Carson J. Holden D. W. Lucia S. M. Dinauer M. C.. (2000). Salmonella pathogenicity island 2-dependent evasion of the phagocyte NADPH oxidase. Science 287, 1655–1658. 10.1126/science.287.5458.1655 10698741

[B98] Wallis T. S. Paulin S. M. Plested J. S. Watson P. R. Jones P. W. (1995). The Salmonella dublin virulence plasmid mediates systemic but not enteric phases of salmonellosis in cattle. Infect. Immun. 63, 2755–2761. 7790094 10.1128/iai.63.7.2755-2761.1995 PMC173368

[B99] Wang Y. Disalvo M. Gunasekara D. B. Dutton J. Proctor A. Lebhar M. S. (2017). Self-renewing monolayer of primary colonic or rectal epithelial cells. Cell. Mol. Gastroenterol. Hepatol. 4, 165–182.e167. 10.1016/j.jcmgh.2017.02.011 29204504 PMC5710741

[B100] Watson P. R. Galyov E. E. Paulin S. M. Jones P. W. Wallis T. S. (1998). Mutation of invH, but not stn, reduces Salmonella-induced enteritis in cattle. Infect. Immun. 66, 1432–1438. 9529064 10.1128/iai.66.4.1432-1438.1998 PMC108071

[B101] Wilson S. S. Bromme B. A. Holly M. K. Wiens M. E. Gounder A. P. Sul Y.. (2017). Alpha-defensin-dependent enhancement of enteric viral infection. PLoS Pathog. 13:e1006446. 10.1371/journal.ppat.1006446 28622386 PMC5489213

[B102] Wilson S. S. Tocchi A. Holly M. K. Parks W. C. Smith J. G. (2015). A small intestinal organoid model of non-invasive enteric pathogen-epithelial cell interactions. Mucosal. Immunol. 8, 352–361. 10.1038/mi.2014.72 25118165 PMC4326599

[B103] Wistuba I. I. Gazdar A. F. (2004). Gallbladder cancer: lessons from a rare tumour. Nat. Rev. Cancer 4, 695–706. 10.1038/nrc1429 15343276

[B104] Wong K. K. Mcclelland M. Stillwell L. C. Sisk E. C. Thurston S. J. Saffer J. D. (1998). Identification and sequence analysis of a 27-kilobase chromosomal fragment containing a Salmonella pathogenicity island located at 92 minutes on the chromosome map of *Salmonella enterica* serovar typhimurium LT2. Infect. Immun. 66, 3365–3371. 9632606 10.1128/iai.66.7.3365-3371.1998 PMC108353

[B110a] Woo J. L. Berk A. J. (2007). Adenovirus ubiquitin-protein ligase stimulates viral late mRNA nuclear export. J. Virol. 81, 575–587. 10.1128/JVI.01725-06 17079297 PMC1797459

[B105] Wood M. W. Jones M. A. Watson P. R. Hedges S. Wallis T. S. Galyov E. E. (1998). Identification of a pathogenicity island required for Salmonella enteropathogenicity. Mol. Microbiol. 29, 883–891. 10.1046/j.1365-2958.1998.00984.x 9723926

[B106] Wray C. Sojka W. J. (1978). Experimental Salmonella typhimurium infection in calves. Res. Vet. Sci. 25, 139–143. 364573

[B107] Yin X. Farin H. F. Van Es J. H. Clevers H. Langer R. Karp J. M. (2014). Niche-independent high-purity cultures of Lgr5+ intestinal stem cells and their progeny. Nat. Methods 11, 106–112. 10.1038/nmeth.2737 24292484 PMC3951815

[B108] Yin Y. Bijvelds M. Dang W. Xu L. Van Der Eijk A. A. Knipping K.. (2015). Modeling rotavirus infection and antiviral therapy using primary intestinal organoids. Antiviral Res. 123, 120–131. 10.1016/j.antiviral.2015.09.010 26408355

[B115a] Yin Y. Dang W. Zhou X. Xu L. Wang W. Cao W.. (2017). PI3K-Akt-mTOR axis sustains rotavirus infection via the 4E-BP1 mediated autophagy pathway and represents an antiviral target. Virulence 9, 83–98. 10.1080/21505594.2017.1326443 28475412 PMC5955461

[B116a] Yin Y. Wang Y. Dang W. Xu L. Su J. Zhou X.. (2016). Mycophenolic acid potently inhibits rotavirus infection with a high barrier to resistance development. Antiviral Res. 133, 41–49. 10.1016/j.antiviral.2016.07.017 27468950

[B109] Young M. Reed K. R. (2016). Organoids as a model for colorectal cancer. Curr. Colorectal Cancer Rep. 12, 281–287. 10.1007/s11888-016-0335-4 27656116 PMC5016547

[B110] Zachos N. C. Kovbasnjuk O. Foulke-Abel J. In J. Blutt S. E. De Jonge H. R.. (2016). Human enteroids/colonoids and intestinal organoids functionally recapitulate normal intestinal physiology and pathophysiology. J. Biol. Chem. 291, 3759–3766. 10.1074/jbc.R114.635995 26677228 PMC4759158

[B111] Zhang K. Dupont A. Torow N. Gohde F. Leschner S. Lienenklaus S.. (2014). Age-dependent enterocyte invasion and microcolony formation by Salmonella. PLoS Pathog. 10:e1004385. 10.1371/journal.ppat.1004385 25210785 PMC4161480

[B112] Zhang Y. G. Wu S. Xia Y. Sun J. (2014). Salmonella-infected crypt-derived intestinal organoid culture system for host-bacterial interactions. Physiol. Rep. 2:e12147. 10.14814/phy2.12147 25214524 PMC4270227

[B113] Zhou D. (2001). Collective efforts to modulate the host actin cytoskeleton by Salmonella type III-secreted effector proteins. Trends Microbiol. 9, 567–569; discussion 569–570. 10.1016/S0966-842X(01)02227-2 11728859

[B114] Zhou D. (2006). Bacterial invasion into non-phagocytic cells, in Molecular Paradigms of Infectious Disease, eds Nickerson C. A. Schurr M. J. (New York, NY: Springer Ecience+Business Media, LLC), 247–273.

[B115] Zhou D. Galán J. (2001). Salmonella entry into host cells: the work in concert of type III secreted effector proteins. Microbes Infect. 3, 1293–1298. 10.1016/S1286-4579(01)01489-7 11755417

[B116] Zhu S. Ding S. Wang P. Wei Z. Pan W. Palm N. W.. (2017). Nlrp9b inflammasome restricts rotavirus infection in intestinal epithelial cells. Nature 546, 667–670. 10.1038/nature22967 28636595 PMC5787375

[B117] Zierler M. K. Galán J. E. (1995). Contact with cultured epithelial cells stimulates secretion of Salmonella typhimurium invasion protein InvJ. Infect. Immun. 63, 4024–4028. 7558314 10.1128/iai.63.10.4024-4028.1995 PMC173565

[B126a] Zou W. Y. Blutt S. E. Crawford S. E. Ettayebi K. Zeng X. L. Saxena K.. (2017). Human intestinal enteroids: new models to study gastrointestinal virus infections. Methods Mol. Biol. 10.1007/7651_2017_1. [Epub ahead of print]. 28361480 PMC5752619

