# Corrigendum: Organoid and Enteroid Modeling of *Salmonella* Infection

**DOI:** 10.3389/fcimb.2018.00257

**Published:** 2018-07-27

**Authors:** Yuebang Yin, Daoguo Zhou

**Affiliations:** ^1^Department of Gastroenterology and Hepatology, Erasmus MC-University Medical Center, Rotterdam, Netherlands; ^2^Key Laboratory of Molecular Microbiology and Technology, Ministry of Education, TEDA Institute of Biological Sciences and Biotechnology, Nankai University, Tianjin, China; ^3^Department of Biological Sciences, Purdue University, West Lafayette, IN, United States

**Keywords:** *Salmonella*, infection models, enteroids, intestine, organoids

In the original article several articles were cited in Tables 1 and 2, but were not included in the Reference list. The following articles appear in the reference list below:

Barrila, J., Radtke, A. L., Crabbe, A., Sarker, S. F., Herbst-Kralovetz, M. M., Ott, C. M., et al. (2010). Organotypic 3D cell culture models: using the rotating wall vessel to study host-pathogen interactions. *Nat. Rev. Microbiol*. 8, 791–801. doi: 10.1038/nrmicro2423

Bartfeld, S., and Clevers, H. (2015). Organoids as model for infectious diseases: culture of human and murine stomach organoids and microinjection of *Helicobacter pylori*. *J. Vis Exp*. doi: 10.3791/53359. [Epub ahead of print].

Boyle, E. C., Dombrowsky, H., Sarau, J., Braun, J., Aepfelbacher, M., Lautenschläger, I., et al. (2015). *Ex vivo* perfusion of the isolated rat small intestine as a novel model of Salmonella enteritis. *Am. J. Physiol. Gastrointest. Liver Physiol*. 310, G55–G63. doi: 10.1152/ajpgi.00444.2014

Dostal, A., Gagnon, M., Chassard, C., Zimmermann, M. B., O'mahony, L., and Lacroix, C. (2014). Salmonella adhesion, invasion and cellular immune responses are differentially affected by iron concentrations in a combined *in vitro* gut fermentation-cell model. *PLoS ONE* 9:e93549. doi: 10.1371/journal.pone.0093549

Mathur, R., Oh, H., Zhang, D., Park, S. G., Seo, J., Koblansky, A., et al. (2012). A mouse model of salmonella typhi infection. *Cell* 151, 590–602. doi: 10.1016/j.cell.2012.08.042

Woo, J. L., and Berk, A. J. (2007). Adenovirus ubiquitin-protein ligase stimulates viral late mRNA nuclear export. *J. Virol*. 81, 575–587. doi: 10.1128/JVI.01725-06

Yin, Y., Dang, W., Zhou, X., Xu, L., Wang, W., Cao, W., et al. (2017). PI3K-Akt-mTOR axis sustains rotavirus infection via the 4E-BP1mediated autophagy pathway and represents an antiviral target. *Virulence* 9, 83–98. doi: 10.1080/21505594.2017.1326443

Yin, Y., Wang, Y., Dang, W., Xu, L., Su, J., Zhou, X., et al. (2016). Mycophenolic acid potently inhibits rotavirus infection with a high barrier to resistance development. *Antiviral Res*. 133, 41–49. doi: 10.1016/j.antiviral.2016.07.017

Zou, W. Y., Blutt, S. E., Crawford, S. E., Ettayebi, K., Zeng, X. L., Saxena, K., et al. (2017). Human intestinal enteroids: new models to study gastrointestinal virus infections. *Methods Mol. Biol*. doi: 10.1007/7651_2017_1. [Epub ahead of print].

The authors apologize for this error and state that this does not change the scientific conclusions of the article in any way.

The original article has been updated.

## Conflict of interest statement

The authors declare that the research was conducted in the absence of any commercial or financial relationships that could be construed as a potential conflict of interest.

